# Latitudinal Clines of the Human Vitamin D Receptor and Skin Color Genes

**DOI:** 10.1534/g3.115.026773

**Published:** 2016-02-26

**Authors:** Dov Tiosano, Laura Audi, Sharlee Climer, Weixiong Zhang, Alan R. Templeton, Monica Fernández-Cancio, Ruth Gershoni-Baruch, José Miguel Sánchez-Muro, Mohamed El Kholy, Zèev Hochberg

**Affiliations:** * Division of Pediatric Endocrinology, Mayer Children’s Hospital, Rambam Medical Center Haifa 30196, Israel, Haifa, Israel; † Rappaport Family Faculty of Medicine, The Technion - Israel Institute of Technology, Haifa 30196, Israel, Haifa, Israel; ‡ Pediatric Endocrinology, Vall d’Hebron Research Institute (VHIR), CIBERER, Autonomous University, 08035 Barcelona, Spain; § Department of Computer Science and Engineering, Washington University, St. Louis, Missouri; ** Department of Biology, Washington University, St. Louis, Missouri 63130-4899; †† Department of Evolutionary and Environmental Biology, University of Haifa Haifa 39105, Israel; ‡‡ Department of Human Genetics, Rambam Medical Center, Haifa, Israel; §§ Pediatric Service, Area Basica de Salut (ABS), Salt, 17001 Girona, Spain; *** Department of Pediatrics, Ain Shams University, 11566 Cairo, Egypt

**Keywords:** epistasis, skin color, vitamin D, linkage disequilibrium, network analysis, adaptation

## Abstract

The well-documented latitudinal clines of genes affecting human skin color presumably arise from the need for protection from intense ultraviolet radiation (UVR) *vs.* the need to use UVR for vitamin D synthesis. Sampling 751 subjects from a broad range of latitudes and skin colors, we investigated possible multilocus correlated adaptation of skin color genes with the vitamin D receptor gene (*VDR*), using a vector correlation metric and network method called BlocBuster. We discovered two multilocus networks involving *VDR* promoter and skin color genes that display strong latitudinal clines as multilocus networks, even though many of their single gene components do not. Considered one by one, the *VDR* components of these networks show diverse patterns: no cline, a weak declining latitudinal cline outside of Africa, and a strong in- *vs.* out-of-Africa frequency pattern. We confirmed these results with independent data from HapMap. Standard linkage disequilibrium analyses did not detect these networks. We applied BlocBuster across the entire genome, showing that our networks are significant outliers for interchromosomal disequilibrium that overlap with environmental variation relevant to the genes’ functions. These results suggest that these multilocus correlations most likely arose from a combination of parallel selective responses to a common environmental variable and coadaptation, given the known Mendelian epistasis among *VDR* and the skin color genes.

Linkage disequilibrium (LD) is one approach for the measurement of population-level associations between polymorphic states at two nonhomologous sites. Many LD measures exist, but almost all of them share one feature in common: they are scalars. This means that although there are many different possible gametic combinations, all of this combinatorial diversity is collapsed into a single number. However, under many evolutionarily realistic situations, the association between two loci or nucleotide sites is primarily affected by just a subset of the possible allelic combinations. For example, when a nucleotide site undergoes a mutation to a novel nucleotide state, the LD created by mutation is dominated by the specific combination of the new allele at the mutated site with the specific alleles at all polymorphic sites that happened to be on the same DNA molecule at the time of mutation. Because the association of a newly mutated allele occurs with alleles at multiple preexisting polymorphic sites, the problem of association is also inherently of high dimension and not limited to just two loci or nucleotide positions (Smouse 1974). Another example is when selection favors one particular combination of alleles over others due to fitness epistasis, which can also involve more than two loci. In these and other cases, a subset of specific allelic combinations at multiple sites or loci are responsible for the associations found between each pair of loci or nucleotide sites, with the remaining allelic combinations making little to no contribution to overall LD. When such genetic heterogeneity exists, a scalar measure of LD loses biological information and results in a loss of statistical power by pooling the effects of allelic combinations with strong associations with combinations showing little or no association.

To avoid these deficiencies of scalar LD measures, Climer *et al.* (2014b) introduced the Custom Correlation Coefficient (*CCC*), which measures LD as a vector, with separate elements in the vector measuring the association of a specific allelic combination. Because multilocus/site associations are not limited just to pairs of loci or SNPs (single nucleotide polymorphisms) (Smouse 1974), Climer *et al.* (2014a) coupled *CCC* with a program called BlocBuster, which extracts all the significant *CCC* elements and interconnects multiple loci or SNPs through shared allelic nodes. By interconnecting alleles at multiple loci into a network, BlocBuster avoids the problem of looking at associations only at pairs of loci or nucleotides. By exhaustively scanning all pairs of loci or SNPs while constructing these allelic networks, BlocBuster avoids another problem in examining multilocus associations: homoplasy. Homoplasy occurs when multiple mutations occur at the same locus or site such that the same allelic state arises more than once; that is, alleles are identical-by-state by not identical-by-descent. Similarly, gene conversion can paste a preexisting allelic state onto a new chromosomal background, generating the appearance of homoplasy in a new haplotype. Most mutations and gene conversion events in the human genome are highly clustered into hotspots (Templeton *et al.* 2000a), making homoplasy quite common. For example, 51% of the variable SNP sites in a 9.7 kb region within the Lipo-Protein Lipase gene resequenced in a sample of 71 humans displayed homoplasy, even after apparent homoplasies due to recombination or gene conversion had been eliminated (Templeton *et al.* 2000b). Such homoplasy can weaken associations between sites, particularly for scalar measures, and can interfere with many algorithms for reconstructing haplotypes. By using *CCC* and BlocBuster, such mutational and gene conversion hotspots can either be passed over in constructing a haplotype network and/or the vector elements measuring specific allelic associations not affected by homoplasy can be identified and separated from those affected by homoplasy.

The strength of BlocBuster is illustrated by recent work on the *Gephyrin* locus using publically available HapMap data. Park (2012) used HapMap Phase III genotype data to survey patterns of LD in the human genome using scalar measures and found an exceptionally strong LD block in the *Gephyrin* region. Climer *et al.* (2015) performed a BlocBuster survey of the human genome using HapMap Phase III genotype data and discovered that the LD in this region is caused by extensive multisite associations, involving two haplotypes that differ by 284 divergent nucleotide states with almost no intermediate haplotypes present. Such highly divergent haplotype pairs that represent the majority of the existing haplotypes are called “yin-yang” pairs (Zhang *et al.* 2003). The *Gephyrin* haplotypes are the longest yin-yang pair ever found in the human genome by an order of magnitude, yet this dramatic yin-yang structure had been overlooked in data readily available to the scientific community. The reason is that BlocBuster not only detects LD and multi-site associations but, by making it allele-specific, it also can phase genotype data into haplotypes. Much more biological information is therefore available in a BlocBuster analysis than in a scalar LD analysis, revealing in this case a dramatic pattern in the human genome that was invisible to scalar LD measures.

BlocBuster can deal with all pairwise *CCC* measures of SNPs in the human genome, not just those that are tightly linked. For example, in a case/control study on hypertensive heart disease, BlocBuster identified a network of 25 SNPs, 13 of which defined a haplotype in the *SLC8AI* gene that is involved in returning the heart to a resting state after excitation, with the remaining SNPs spanning six additional genes on five different chromosomes, all different from the chromosome on which *SLC8AI* is located (Climer *et al.* 2014b). Because this was a case/control study, the associations between unlinked genes may reflect the genetic architecture of the phenotype under study rather than interchromosomal LD in the general population, because the “case” portion of the sample was obtained nonrandomly on the basis of a phenotype that is uncommon in the general population.

We wanted to see if *CCC* and BlocBuster could identify population-level associations between unlinked genes that are statistically significant, biologically informative, and evolutionarily important, but that are invisible to scalar measures of LD. Here, we focus on a set of unlinked candidate genes involved in human skin pigmentation and vitamin D metabolism. Many of the skin pigmentation genes display latitudinal clines that are thought to arise from two selective forces related to ultraviolet B radiation (UVB): selection in high UVB environments for dark, photoprotective, eumelanin-rich pigmentation, and selection in low UVB environments for light pigmentation arising from the requirement for UVB in sunlight to sustain cutaneous photosynthesis of vitamin D3 (Jablonski and Chaplin 2010). Several genome-wide association studies have identified single nucleotide polymorphism (SNP) markers in genes associated with skin color variation (Cerqueira *et al.* 2012; Beleza *et al.* 2013a), many of which reveal evidence of positive selection (Jablonski and Chaplin 2010; Beleza *et al.* 2013a). In addition, we include the Vitamin D Receptor (*VDR*) gene because *VDR* and the skin color genes jointly influence vitamin D metabolism and pigmentation in a manner characterized by epistasis (Pośpiech *et al.* 2014). Because these genes respond to a common environmental factor and exhibit epistasis, this is a promising candidate set for displaying allele-specific associations across human populations. Specifically, we applied BlocBuster to a genetic survey of 64 SNPs at the *VDR* gene and seven genes associated with skin color from 751 subjects sampled from a range of geographical latitudes and skin colors. The *VDR* gene has not been reported to show latitudinal clines associated with positive selection, so we disproportionately sampled SNPs from *VDR*, including the promoter region. We analyzed these data with traditional LD measures to make a direct comparison with the BlocBuster results. We corroborated our results by analyzing HapMap data on these same genes. Finally, we applied BlocBuster to all the available SNP data on four to eight HapMap populations as a genomic control for associations generated by demographic or historical factors rather than natural selection.

## Materials and Methods

### Populations sampled

DNA samples were collected from 751 subjects from Haifa, Israel; Salt, Spain; and Cairo, Egypt. The ethical review boards of the Haifa-Rambam Health Care Campus, the IDIAP Jordi Gol of Barcelona, and Cairo University approved the protocols, and informed consents were obtained from each adult or legal guardian representing a child. Subjects self-defined for ethnicity as shown in Table 1. Samples taken from individuals no longer living in the area associated with their ethnic origin were the children of parents who were both direct immigrants from the relevant ethnic region. Consequently, there was no opportunity for interethnic genetic mixing in the country of current residence.

**Table 1:** Ethnic distribution of the study subjects

Site of Sample Origin	Ethnic Origin	Number	Approximate Latitude of Ethnic Origin
Haifa, Israel	Western European ancestry Jews	163	50
	Ethiopian Jews	9	8
	Indian Jews - out of Cochin	20	25
	Yemenite Jews	79	16
	Arab Christians	70	32
	Arab Muslims	60	28
Salt, Spain	Spanish Caucasians	83	40
	North Africans from Maghreb	85	34
	Sub-Saharan from different countries	98	8
	South Americans (Amerindian)	20	15
	India	14	25
Cairo, Egypt	Egyptians	50	30

Because of small sample sizes, the Ethiopian Jews were pooled with the other sub-Saharan populations, and the Indian Jews were pooled with the other Indian samples (there were no significant frequency differences in any of the networks identified), yielding a total of 10 populations. Latitudes were assigned to populations based on a central locality corresponding to their ethnic origin. This could reduce the geographical accuracy of the latitudinal assignments, which would reduce our statistical power to detect latitudinal associations by introducing potential error. Hence, our analyses of latitudinal associations are conservative from a statistical perspective.

### Genotyping

SNPs within the *VDR* gene and within skin color genes (*TYR*, *TYRP1*, *OCA2*, *SLC45A2*, *SLC24A5*, *KITLG* and *MC1R*) were selected to fill a 64-SNP array as indicated in Supplemental Material, Table S1. Genotyping was performed at a central laboratory (L.A. and M.F.-C.) using Applied Biosystems Taqman and OpenArray technologies (Life Technologies, Carlsbad). SNPs were typed using TaqMan genotyping chemistry supported on a metal-based array. DNA samples were loaded and amplified on arrays as recommended by the manufacturer. Arrays were scanned on the OpenArray NT imager and genotypes were called using the OpenArray SNP Genotyping analysis software. All of the eight loci we surveyed were on different chromosomes; so interlocus was the same as interchromosomal in all of our analyses.

### Analytical methods

#### Custom correlation coefficient:

We measured the associations between SNP pairs with the *CCC*, which returns a vector, with the vector elements measuring the degree of association between specific allelic pairs (Climer *et al.* 2014b). For two biallelic SNPs (the only type analyzed in our data set), the formula for the *t*^th^ element of the *CCC* vector is:

$$CCC_{ij}\left[t\right]=\left(9/2\right)R_{ij}\left[t\right]ff_{it}ff_{jt}$$ (1)

where *t* represents an index number for one of the possible allelic combinations at SNP *i* and SNP *j* (*e.g.*, if *A* and *a* are the alleles at SNP *i*, and *B* and *b* are the alleles at SNP *j*, then *t* has four values for the combinations *AB*, *Ab*, *aB*, *ab*), (9/2) is an empirically derived scaling factor to make the *CCC* values lie between 0 and 1 (with no impact on any statistical inferences), *R_ij_*[*t*] is the average contribution across the individuals that have no missing data for either SNP to the frequency of the *t*^th^ allelic combination, and *ff_kt_* is a frequency factor to account for the impact of the frequency of the allele at SNP *k* upon observing allelic combination *t* that contains this allele. A simple factor *ff_kt_* = 1 – *f_kt_* / *q*, is utilized, where *f_kt_* is the frequency of the relevant allele at SNP *k* from allelic combination *t*, and *q* is a weighting factor with a default value of 1.5 (for more details, see Climer *et al.* 2014b). Several examples of these calculations are given in Climer *et al.* (2014b), so we only provide one here. Suppose a sample of 10 individuals was taken and scored for two biallelic SNPs with the following results: one individual was *Aa/bb*, one individual was *AA/Bb*, and eight individuals were *AA/bb*. Then the vector *R_12_* = (0.05, 0.90, 0.00, 0.05) for the four allelic combinations *AB*, *Ab*, *aB*, and *ab* respectively, and the vector *CCC_12_* = (0.08, 0.54, 0.00, 0.08), indicating a strong association between the alleles *A* and *b*.

Missing data are common in SNP data sets, and typically this is corrected with imputation that uses knowledge about LD patterns from previous data sets. However, imputation biases the results toward inflated LD. Also, we were surveying many populations that have little previous data, which makes imputation even more error-prone (Jewett *et al.* 2012). Consequently, we omitted individuals with missing or imputed genotypes for either of the SNPs in the pair.

The *CCC* values were calculated for all SNP allelic pairs from all individuals in the total data set without regard to population of origin, linkage relationships, or any latitudinal information. The significance of a *CCC* value and false positive rates were assessed through 1,000,000 permutation trials, in which the genotype states were randomly permuted across all individuals in the sample to simulate the null hypothesis of no association between all pairs of loci and alleles across and within all populations. The permutation tests were done over all possible 2016 SNP pairs, and the maximum *CCC* values over all pairs were determined for each trial. This set of maximum values determines the false-positive thresholds under the null hypothesis of no associations.

#### BlocBuster:

Our open-source code is available at www.blocbuster.org or by contacting the author S. Climer. BlocBuster (Climer *et al.* 2014a) is a deterministic network-based, genome-wide association analysis method that employs *CCC* (Climer *et al.* 2014b). A BlocBuster network is constructed with 2*n* nodes, where *n* is equal to the number of SNPs. Each SNP is represented by two nodes, one for each of its alleles (the current implementation assumes biallelic SNPs). BlocBuster calculates *CCC* vectors for all pairs of SNPs, and then creates allele-specific networks by joining those alleles that have a *CCC* element value that is greater than a threshold determined by permutation testing. This threshold will be sensitive to both the sample size and the number of SNPs being surveyed. In particular, as sample size increases, we would expect the threshold to decrease because of increased statistical power. In contrast, the threshold is expected to increase as the number of SNPs surveyed increases because of the need to correct for more pairwise comparisons. Hence, the threshold value determined by permutation testing will in general vary from dataset to dataset.

BlocBuster transforms the allele-specific pairs with significant associations (at or above the threshold) into higher-ordered relationships by joining together all pairs that share an allele in common. We have observed that BlocBuster results built from biological data consist of large numbers of singletons (a SNP allele with no significant connections to any other allele) and discrete clusters of networks with relatively high densities of edges. These networks naturally separate the heterogeneity within the mixture of all populations, yielding potentially significant frequency variations between distinct populations. In this manner, networks of alleles showing strong associations arise automatically through BlocBuster with no need for an additional clustering algorithm.

#### Analysis of networks:

The frequency of individuals included within a particular BlocBuster network was determined for each population. Least-squares regressions of network population frequencies against the latitudes of the populations were implemented with JMP 5.0.1 (SAS Institute Inc.) using the sample sizes as weights. The JMP software uses the Gauss-Markov theorem to orthogonalize the regression vector space, and all the estimated regression coefficients are for the orthogonalized space. These orthogonalized coefficients do not necessarily correspond to the slopes and intercepts plotted in a Cartesian space. Because of missing data, the sample sizes varied across the BlocBuster networks. Because there is no prior information available about how these networks could influence fitness through the environmental variable of UVB, we have no prior expectation of the type of relationship between network frequency and latitude. We therefore performed both linear and quadratic regressions against latitude, as there are not enough distinct latitude points to justify higher-order regressions or more highly parameterized models. If the quadratic term was not significant, we only report the results from the linear regression. In every case in which there was a significant association with latitude, these simple linear and quadratic models explained a majority of the variation, as measured by R^2^. JMP was also used to test the null hypothesis of no frequency differences across various groups of populations, such as sub-Saharan Africans *vs.* all other populations.

#### LD analysis:

The r^2^ and D’ LD values were computed between all 2016 pairs of our 64 SNPs using Plink (Purcell *et al.* 2007), available at http://pngu.mgh.harvard.edu/~purcell/plink/ld.shtml. The “Pairwise LD measures for multiple SNPs (genome-wide)” option was selected. The statistical significances of the LD measures were determined by 1000 permutation trials and computing r^2^ and D’ on the permuted data using Plink. To correct for multiple testing, we first excluded those SNP pairs for which r^2^ or D’ could not be calculated by Plink because of the sparseness of the data (261 pairs in the original matrices). Next, given that the LD values are not independent and are further constrained by the estimation of allele frequencies, we used the step-down, stepwise, resampling method with enforced monotonicity, which incorporates constrained and correlated structures (Westfall and Young 1993) to achieve an overall type I error rate of 5% separately for r^2^ and D’.

In order to concentrate the statistical power on the interchromosomal pairs of SNPs, we repeated the analysis by permuting intact, multi-SNP genotypes at each gene; that is, we used genes as the units of permutation rather than SNPs. By permuting intact genes, we can no longer test intragenic disequilibrium, so all the statistically relevant contrasts are for the interchromosomal SNP pairs. The step-down procedure was used to adjust type I error rate to 5% for the unlinked SNP LD values.

#### HapMap data supplement:

To confirm our results with a second data set and to control for possible demographic effects causing associations at the genome level, we downloaded bulk HapMap data from http://hapmap.ncbi.nlm.nih.gov/, using the release: HapMap r28, nr.b36 dated 18-Aug-2010 from directory /downloads/genotypes/2010-08_phaseII+III/forward/. The HapMap populations used and numbers of individuals are shown in Table 2. Latitudes were assigned to a central location to these populations according to ethnic origin. We built the BlocBuster networks using the CEU, CHB, JPT, and YRI data, the populations with the most SNPs. Some of the individuals were related, as tabulated here: http://hapmap.ncbi.nlm.nih.gov/downloads/samples_individuals/relationships_w_pops_121708.txt. After removing the children from the CEU and YRI data, we had a total of 490 unrelated individuals. BlocBuster was applied to all 1,115,561 autosomal SNPs scored in these four populations. We extracted the BlocBuster analysis on 571 HapMap SNPs in the four genes and their promoter regions that define network 65_2 (*VDR*, *MC1R*, *SLC24A5*, and *SLC45A2*), the BlocBuster network yielding the strongest evidence for multilocus latitudinal association. Based upon 1000 permutation trials, the *CCC* threshold was set to 0.84 to eliminate false positives. No false positives were detected in any of the 1000 trials at this threshold. To investigate the impact of the threshold value on the results, we repeated this analysis at threshold values of 0.80, 082, and 0.86, in addition to the 0.84 threshold.

**Table 2:** The HapMap populations used in this study

Population	Description	Number of Individuals
CEU	Utah residents with Northern and Western European ancestry from the CEPH collection	116
YRI	Yoruba in Ibadan, Nigeria	119
JPT	Japanese in Tokyo, Japan	116
CHB	Han Chinese in Beijing, China	139
GIH	Gujarati Indians in Houston, Texas	101
TSI	Toscans in Italy	102
LWK	Luhya in Webuye, Kenya	110
MKK	Maasai in Kinyawa, Kenya	143

To provide a broader range of latitudes, we analyzed four additional HapMap populations: GIH, LWK, MKK, and TSI. After removing MKK children, we had a total of 456 unrelated individuals. By using the SNPs held in common between those listed in Table S1 and the HapMap data, we calculated the frequencies in all eight of the HapMap populations of partial networks defined originally by the BlocBuster analysis of the 64 SNPs (listed in Table 2 and Table S1) and the BlocBuster analysis of the 571 SNPs in *VDR*, *MC1R*, *SLC24A5*, and *SLC45A2* in the four HapMap populations CEU, CHB, JPT, and YRI. The networks in each case were partial because the additional four HapMap populations were not scored for as many SNPs, and we used only those SNPs that were scored in all the populations.

Another possible data source is the 1000 genomes project (http://www.1000genomes.org/). However, imputation was used extensively in creating these data, and as the website warns, “… we are unable to precisely identify which sites used imputation to generate their genotype.” Because of the biases and errors that imputation can cause for our type of analysis, and because of the inability to identify and exclude the imputed nucleotides, we did not use the 1000 genomes data.

### Data availability

All the SNP genotypes used in our study are available in File S1. The HapMap data we used is available from http://hapmap.ncbi.nlm.nih.gov/, using the release: HapMap r28, nr.b36 dated 18-Aug-2010 from directory /downloads/genotypes/2010-08_phaseII+III/forward/. The ancestral alleles used in our analysis are given in dbSNP, http://www.ncbi.nlm.nih.gov/projects/SNP/index.html. The Neandertal and Denisovan genome databases used are available at http://neandertal.ensemblgenomes.org/ and http://www.eva.mpg.de. Our open-source code for BLOCBUSTER is available at www.blocbuster.org. 

## Results

### BlocBuster applied to 64 candidate SNPs

BlocBuster was applied to 10 populations (Table 1), scored for 64 candidate SNPs (Table S1) that were chosen to have a wide range of latitudes to aid in our search for latitudinal associations. Permutation testing revealed that false positive *CCC* values first appeared at a threshold of 0.65, but even then at an extremely low rate of 0.137 false positives per trial (that is, a total of 136,623 *CCC* elements ≥ 0.65 over 1,000,000 permutation trials). Since all SNPs were biallelic, each pair had four elements in the *CCC* vector, for a total of 8064 *CCC* elements per trial. Hence, the probability of a single *CCC* value exceeding 0.65 by random chance is 0.137 / 8064 = 1.69 × 10^−5^. Because the 8064 pairwise contrasts are not independent, a more conservative way of interpreting the permutation results is with a genome-wide error rate that covers the entire trial and all markers at all loci simultaneously. Of the 1,000,000 permutation trials, the frequency of a complete analysis that included all genes and SNPs without a single false positive *CCC* value using the 0.65 threshold was 0.872398, 0.119015 for one false positive, 0.008153 for two false positives, 0.000434 for three false positives, and 0.000000 for four or more false positives. Hence, any result that yields more than a single *CCC* value at or above the 0.65 threshold can be rejected as being due to chance at the 1% level of significance.

**Figure 1:**
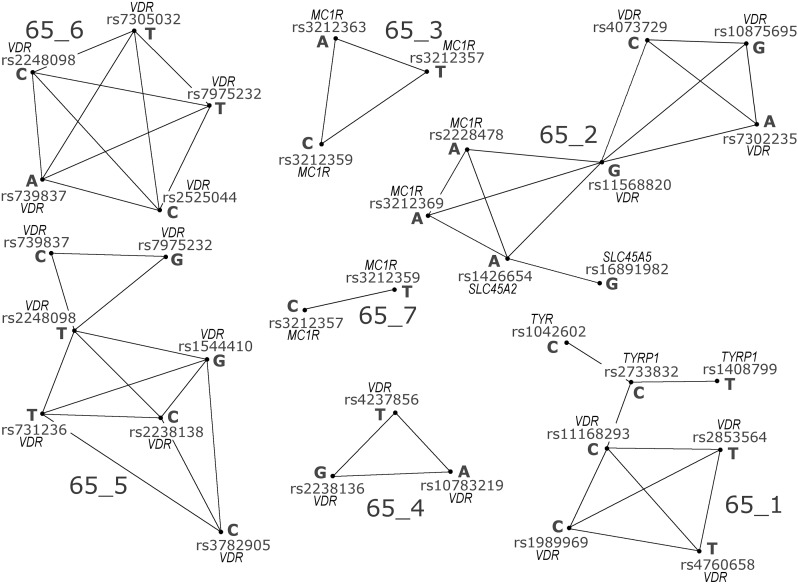
Allelic networks identified by BlocBuster. Each dot represents an allelic node, identified by its SNP and nucleotide state. Out of the total of 128 nodes in the data set, only the nodes that had an edge connecting them to another node are depicted. The 52 edges connecting nodes represent Custom Correlation Coefficient (*CCC*) values ≥ 0.65. These 52 edges defined seven discrete networks of alleles, labeled 65_1 through 65_7.

**Figure 2:**
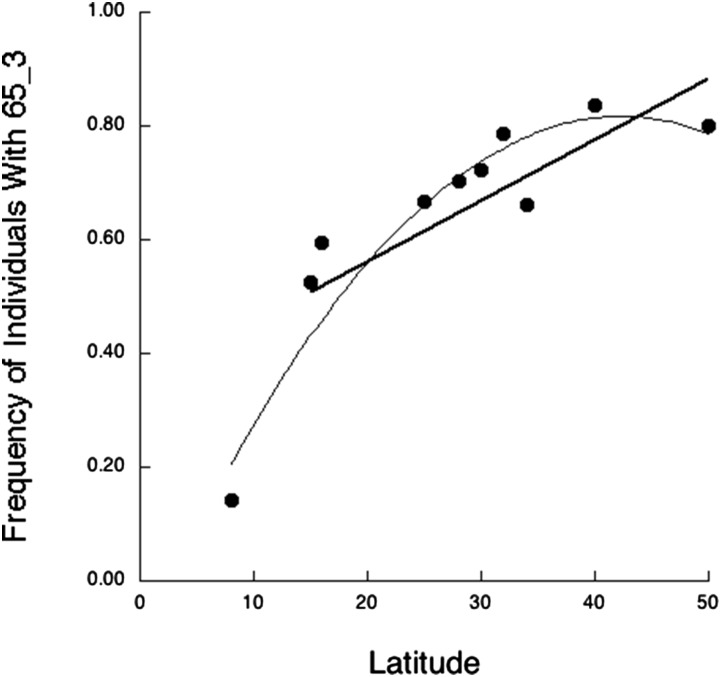
Significant latitudinal clines for haplotype 65_3 identified by BlocBuster in the skin color gene *MC1R*. Haplotype 65_3 shows a significant nonlinear regression with latitude due to the low frequency of this haplotype in sub-Saharan Africa, but exclusion of the sub-Saharan population still results in a significant linear regression with latitude, as shown by the thick straight line.

Using a 0.65 threshold, we obtained seven discrete networks (Figure 1) with a total of 52 *CCC* values ≥ 0.65. Five networks consist of tightly linked SNPs that define haplotypes within a single gene: two haplotypes (65_3 and 65_7) in *MC1R* and three haplotypes (65_4, 65_5, and 65_6) in *VDR* (Table 3). The 65_3 *MC1R* haplotype showed a significant regression of frequency against latitude (Figure 2 and Table 4, with the frequency data given in Table 5), but none of the three *VDR* haplotypes showed any significant association with latitude (Table 4).

**Table 3:** Networks identified by BlocBuster using a threshold of CCC ≥ 0.65 on the samples described in Table 1 and the SNPs described in File S1

Network	Gene	Network ID	SNP	Allele	Chr	Gene Location
65_1	*TYRP1*	70_1	rs2733832	C	9	Intron 5
			rs1408799	T		5′UTR
	*VDR*	70_5	rs4760658	T	12	Promoter (Intron 1a)
			rs11168293	C		
			rs2853564	T		
			rs1989969	C		
	*TYR*	n/a	rs1042602	C	11	Ser192Tyr
65_2	*VDR*	70_4	rs11568820	G	12	Promoter (Cdx2)
			rs10875695	G		Promoter (Intron 1a)
			rs7302235	A		Promoter (Intron 1a)
	*VDR*	n/a	rs4073729	C	12	Promoter
	*MC1R*	n/a	rs2228478	A	16	Thr314Thr
	*MC1R*	n/a	rs3212369	A	16	3′UTR
	*SLC24A5*	n/a	rs1426654	A	15	Thr111Ala
	*SLC45A2*	n/a	rs16891982	G	5	Phe374Leu
65_3	*MC1R*	70_3	rs3212357	T	16	5′UTR
			rs3212359	C		5′UTR
	*MC1R*	n/a	rs3212363	A	16	5′UTR
65_4	*VDR*	n/a	rs4237856	T	12	Promoter
	*VDR*	n/a	rs10783219	A	12	Promoter (Intron 1a)
	*VDR*	n/a	rs2238136	G	12	Promoter (Intron 1a)
65_5	*VDR*	70_6	rs2248098	T	12	Intron 3
			rs1544410	G		Intron 8
			rs731236	T		Exon 9
			rs3782905	C		Intron 2
			rs2238138	C		Intron 2
	*VDR*	70_8	rs739837	C	12	3′ UTR
			rs7975232	G		Intron 8
65_6	*VDR*	70_7	rs7305032	T	12	Intron 5
			rs7975232	T		Intron8
			rs2525044	C		Intron6
			rs739837	A		3′ UTR
	*VDR*	n/a	rs2248098	C	12	Intron 3
65_7	*MC1R*	70_2	rs3212357	C	16	5′UTR
	*MC1R*		rs3212359	T		5′UTR

Components at a threshold of CCC ≥ 0.70 are indicated by 70_x in the Network ID column. SNP, single nucleotide polymorphism; Chr, chromosome; UTR, untranslated region; Ser, serine; Tyr, tyrosine; Thr, threonine; Ala, alanine; Phe, phenylalanine; Leu, leucine.

**Table 4:** The least-squares regressions of frequencies of individuals bearing a specified network or SNP *vs.* latitude for the populations indicated in Table 1 from the frequency and sample size data given in Table 4

Network/SNP	Genes	Intercept	Slope	Quadratic Term	R^2^
65_1	*TYRP1*, *TYR*, *VDR*	0.752	–0.0074**	n.s.	0.70
70_1	*TYRP1*	0.977	–0.0082***	n.s.	0.76
rs1042602	*TYR*	0.890	0.0065*	n.s.	0.56
70_5	*VDR*	0.848	n.s.	–0.0002*	0.53
70_5–Af	*VDR*	0.920	–0.0027**	n.s.	0.68
65_2	*VDR*, *MC1R*, *SLC24A5*, *SLC45A2*	−0.066	0.0200***	n.s.	0.84
70_4	*VDR*	0.608	0.0104*	–0.0007*	0.73
70_4–Af	*VDR*	0.858	n.s.	n.s.	0.15
rs4073729	*VDR*	0.858	n.s.	–0.0002*	0.50
rs4073729–Af	*VDR*	0.942	–0.0023*	n.s.	0.61
rs2228478	*MC1R*	0.741	0.0066**	–0.0005**	0.85
rs2228478–Af	*MC1R*	0.877	n.s.	n.s.	0.21
rs3212369	*MC1R*	0.575	0.0063**	n.s.	0.59
rs1426654	*SLC24A5*	0.579	0.0130**	–0.0007*	0.80
rs1426654–Af	*SLC24A5*	0.810	n.s.	n.s.	0.24
rs16891982	*SLC45A2*	−0.136	0.0209***	n.s.	0.86
65_3	*MC1R*	0.363	0.0125***	–0.0005**	0.91
65_3–Af	*MC1R*	0.511	0.0064**	n.s.	0.69
65_4	*VDR*	0.983	n.s.	n.s.	0.29
65_5	*VDR*	0.462	n.s.	n.s.	0.36
65_6	*VDR*	0.671	n.s.	n.s.	0.00
65_7	*MC1R*	0.728	n.s.	n.s.	0.06

* Significant with *P* ≤ 0.05, ** significant with *P* ≤ 0.01, *** significant with *P* ≤ 0.001. SNP, single nucleotide polymorphism; n.s., not significant; –Af, the sub-Saharan African populations were excluded from the regression analysis.

**Table 5:** The frequency of individuals possessing the SNP allele networks identified with the 0.65 threshold in the 10 sampled populations

Population	65_1	65_2	65_3	65_4	65_5	65_6	65_7
Sub-Saharan	0.667 (99)	0.021 (95)	0.142 (106)	1.000 (78)	0.420 (69)	0.620 (71)	0.877 (106)
Yamane	0.662 (71)	0.278 (72)	0.595 (74)	0.897 (68)	0.629 (35)	0.721 (68)	0.892 (74)
Egypt	0.571 (49)	0.273 (44)	0.723 (47)	0.949 (39)	0.571 (35)	0.703 (37)	0.729 (48)
Arab Muslim	0.510 (51)	0.552 (58)	0.704 (54)	0.895 (57)	0.500 (42)	0.800 (50)	0.727 (55)
Arab Christian	0.453 (64)	0.817 (60)	0.786 (70)	0.966 (59)	0.667 (48)	0.709 (55)	0.600 (70)
Maghreb	0.474 (78)	0.590 (78)	0.662 (80)	0.966 (58)	0.527 (55)	0.607 (56)	0.612 (80)
Spain	0.375 (72)	0.805 (77)	0.836 (73)	0.870 (54)	0.706 (51)	0.722 (54)	0.554 (74)
W. Eur. ancestry	0.413 (143)	0.886 (149)	0.800 (145)	0.873 (150)	0.687 (131)	0.655 (142)	0.574 (148)
India	0.828 (29)	0.344 (32)	0.667 (33)	0.793 (29)	0.792 (24)	0.538 (26)	0.758 (33)
Amerindian	0.667 (18)	0.529 (17)	0.526 (19)	1.000 (13)	0.818 (11)	0.600 (10)	0.750 (20)

Numbers in parenthesis are the sample sizes, which vary across networks due to missing SNP (single nucleotide polymorphism) genotype data.

**Figure 3:**
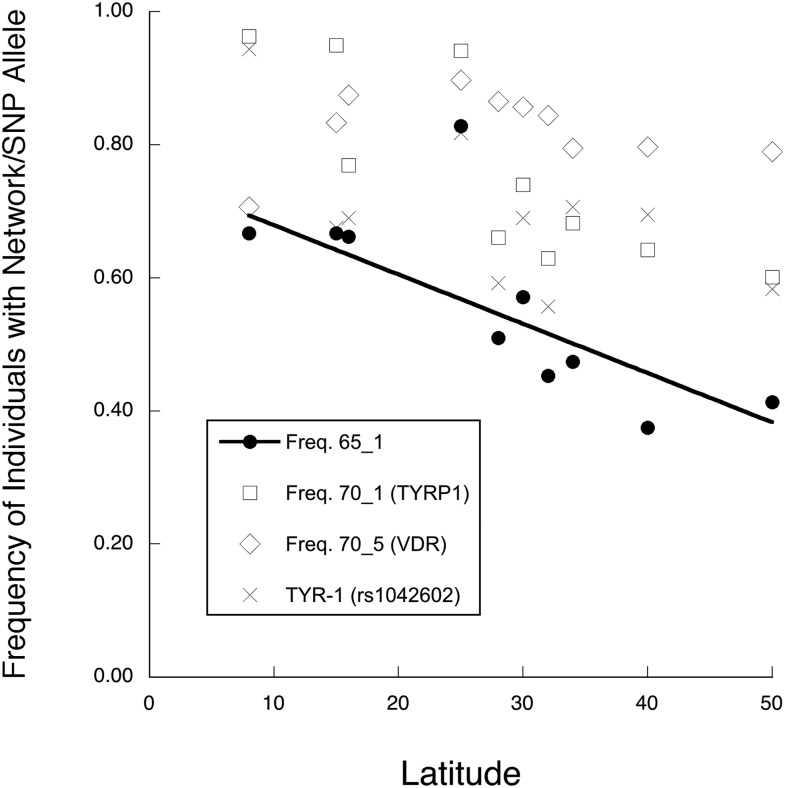
A plot of the frequencies of network 65_1 and its single-gene components *vs.* latitude. The components are the extended haplotype 70_1 in *TYRP1*, the extended promoter haplotype 70_5 in *VDR*, and a single nucleotide polymorphism in *TYR*. The line shows only the regression for the multilocus network 65_1.

**Figure 4:**
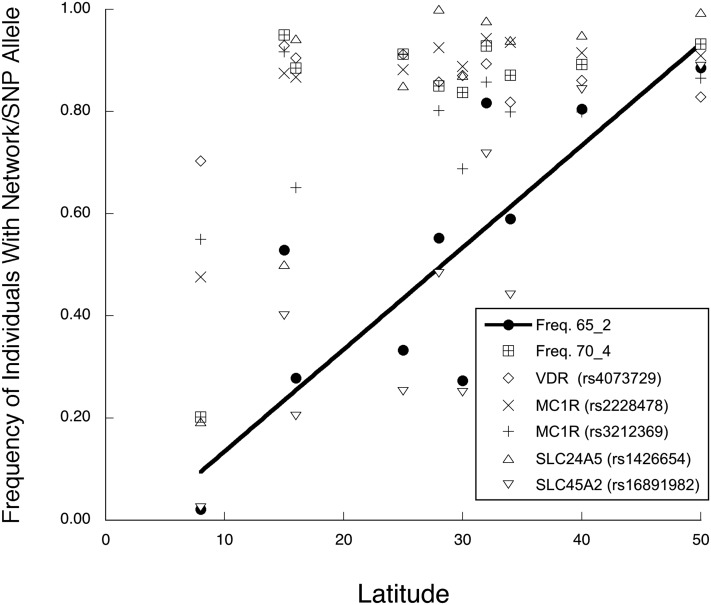
A plot of the frequencies of network 65_2 and its single-gene components *vs.* latitude. The line shows only the regression for the multilocus network 65_2. SNP, single nucleotide polymorphism.

Networks 65_1 and 65_2 (Figure 1) contain SNPs from unlinked genes, with eight intergenic *CCC* values ≥ 0.65. Network 65_1 contains SNPs from *TYRP1*, *TYR*, and *VDR* (Table 3) and displays a significant linear regression with latitude (Figure 3 and Table 4). Network 65_2, consisting of SNPs from *VDR*, *MC1R*, *SLC24A5*, and *SLC45A2* (Table 3), shows an even stronger linear regression against latitude that spans almost the entire frequency range from 0 (near the equator) to 1 (high latitudes) (Figure 4 and Table 4). To gain more insight into these clines, we increased the *CCC* threshold to 0.70, a value at which no false positives occurred in any of the 1,000,000 permutation trials. This higher threshold broke up 65_1 and 65_2 into single locus components (Table 3). The breakup of the network into single-locus components allows us to contrast the latitudinal associations of each single locus component with the latitudinal association of the network as a whole, thereby allowing us to directly see if the whole is simply the sum of the parts.

Network 65_2 gave the strongest cline with latitude. To confirm this result with a second dataset, we attempted to extract the eight SNPs associated with 65_2 from HapMap release 27 for eight HapMap populations with a wide latitudinal spread (Table 2). Unfortunately, no population was scored for all eight of these SNPs in the HapMap data. Indeed, in order to have sufficient populations to investigate latitudinal clines, we could only score four SNPs in three genes in this network*: VDR* rs11568820, *VDR* rs10875695, *MC1R* rs3212369, and *SLC24A5* rs1426654. Hence, the gene *SLC45A2* was dropped. These four SNPs defined a reduced version of 65_2 called 65_2R4, where R4 indicates that the network was reduced by the elimination of four SNPs. The VDR haplotype network 70_4, which is contained within 65_2, was reduced to two VDR SNPs from its original three, which defined the reduced haplotype 70_4R1.

**Figure 5:**
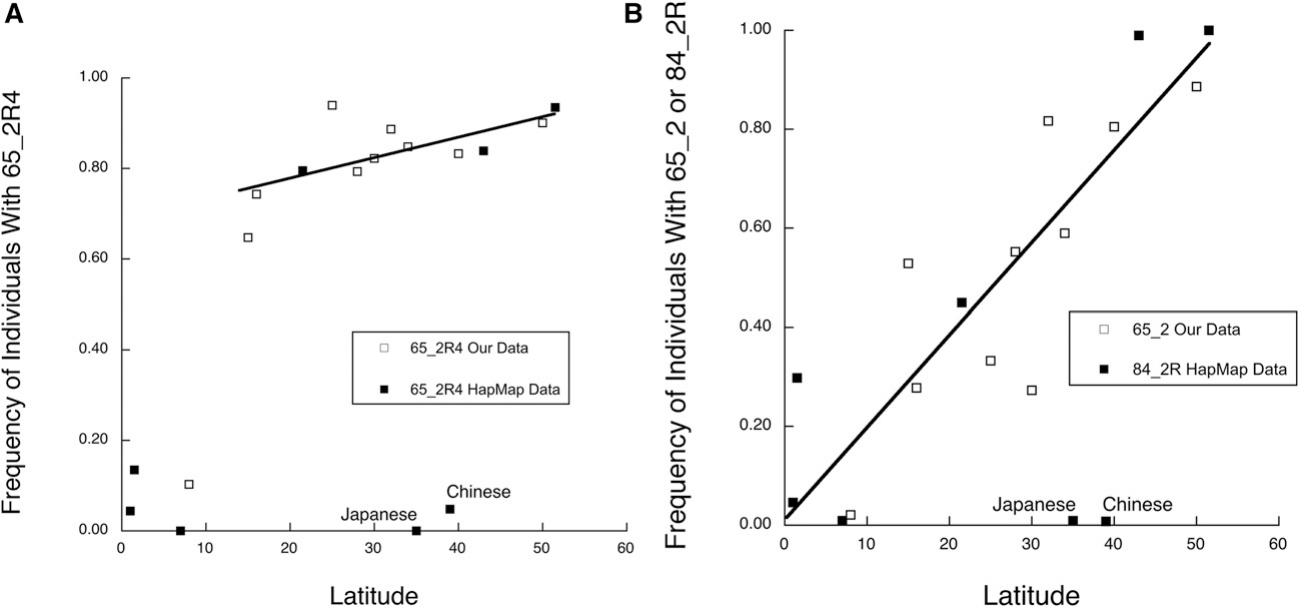
Frequencies of 65_2R4, 65_2, and 84_2 *vs.* latitude. (A) Frequency of individuals with 65_2R4 *vs.* latitude in 10 populations surveyed here and in eight HapMap populations. The line indicates the linear regression when the Han Chinese, Japanese, and sub-Saharan populations are excluded. (B) Frequency of individuals with 65_2 *vs.* latitude in 10 populations surveyed here and with 84_2 in eight HapMap populations. The line indicates the linear regression when the Han Chinese and Japanese populations are excluded.

We calculated the frequency of 65_2R4 in our own data by excluding the SNPs missing in the HapMap data. The frequencies of 65_2R4 in both our data and in the HapMap data interpolate well, with the exception of the Han Chinese and Japanese HapMap populations that represent a geographical area not represented in our own dataset (Figure 5A). These two Asian populations retain a low, African-like frequency of the *VDR* promoter haplotype (Figure 5A), with both 70_4 and 70_4R1 having nearly the same frequency distribution. When these two Asian populations were excluded, our data combined with the HapMap data fitted well to a quadratic regression with a high R^2^ value (a standard measure of fit in regression models that ranges from 0 [poor fit] to 1 [complete fit]) of 0.89 (Table 6). Excluding the sub-Saharan populations in addition to the Chinese and Japanese populations results in a significant linear regression (Table 6).

**Table 6:** The least-squares regressions of frequencies of individuals bearing the reduced networks 70_4R1 and 65_2R4 in our data set and in the HapMap data set

Network/SNP	Dataset	Genes	Intercept	Slope	Quadratic Term	R^2^
70_4R1	Ours	*VDR*	0.600	0.0106*	–0.0007*	0.74
70_4R1 – Af	Ours	*VDR*	0.854	n.s.	n.s.	0.18
70_4R1	HapMap	*VDR*	0.215	0.0148**	n.s.	0.76
70_4R1 – Af	HapMap	*VDR*	0.633	n.s.	n.s.	0.51
65_2R4	Ours	*VDR*, *MC1R*, *SLC24A5*	0.484	0.0129**	–0.0007**	0.85
65_2R4 – Af	Ours	*VDR*, *MC1R*, *SLC24A5*	0.698	0.0042*	n.s.	0.54
65_2R4	HapMap	*VDR*, *MC1R*, *SLC24A5*	0.021	n.s.	n.s.	0.48
65_2R4 – As	HapMap	*VDR*, *MC1R*, *SLC24A5*	0.046	0.0183**	n.s.	0.90
65_2R4 – As	All	*VDR*, *MC1R*, *SLC24A5*	0.271	0.0189***	–0.0005**	0.89
65_2R4 – Af – As	All	*VDR*, *MC1R*, *SLC24A5*	0.693	0.0044**	n.s.	0.65

* Significant with *P* ≤ 0.05, ** significant with *P* ≤ 0.01, *** significant with *P* ≤ 0.001. SNP, single nucleotide polymorphism; n.s., not significant; – Af, the sub-Saharan populations have been excluded; – As, the Chinese and Japanese populations have been excluded.

### LD analysis applied to 64 candidate SNPs

Thirty-two of the pairs of the 64 candidate SNPs in the samples shown in Table 1 had significant LD at the 5% level after correction for multiple testing for r^2^, and 22 pairs had significant LD at the 5% level after correction for multiple testing for D’ (Table 7). The 22 significant D’ values were a subset of the 32 significant r^2^ values. All 32 significant r^2^ values and all 22 significant D’ values involved tightly-linked pairs of SNPs within the same gene. When intact genes were permutated, no significant D’ or r^2^ values were detected for any pair of unlinked SNPs after correcting for type I error, even though this procedure concentrates all statistical power upon the unlinked SNP pairs.

**Table 7:** Pairs of SNPs (SNP1 and SNP2) with significant linkage disequilibrium (r^2^ or D’, with “*” indicating significant and “n.s.” not significant) at the 5% level after correction for multiple testing

SNP1	Gene	Network ID	SNP2	Gene	Network ID	r^2^	D’
rs1408799	*TYRP1*	65_1	rs2733832	*TYRP1*	65_1	*	*
rs3212357	*MC1R*	65_3 & 65_7	rs3212359	*MC1R*	65_3 & 65_7	*	n.s.
rs3212357	*MC1R*	65_3 & 65_7	rs3212363	*MC1R*	65_3	*	n.s.
rs3212359	*MC1R*	65_3 & 65_7	rs3212363	*MC1R*	65_3	*	*
rs2228478	*MC1R*	65_2	rs3212369	*MC1R*	65_2	*	*
rs2228478	*MC1R*	65_2	rs3212371	*MC1R*	None	*	*
rs11568820	*VDR*	65_2	rs10875695	*VDR*	65_2	*	*
rs11568820	*VDR*	65_2	rs7302235	*VDR*	65_2	*	*
rs10875695	*VDR*	65_2	rs7302235	*VDR*	65_2	*	*
rs4760658	*VDR*	65_1	rs11168293	*VDR*	65_1	*	*
rs4760658	*VDR*	65_1	rs2853564	*VDR*	65_1	*	*
rs11168293	*VDR*	65_1	rs2853564	*VDR*	65_1	*	*
rs2853564	*VDR*	65_1	rs1989969	*VDR*	65_1	*	*
rs3782905	*VDR*	65_5	rs2238138	*VDR*	65_5	*	*
rs2248098	*VDR*	65_5 & 65_6	rs7305032	*VDR*	65_6	*	n.s.
rs2248098	*VDR*	65_5 & 65_6	rs2525044	*VDR*	65_6	*	n.s.
rs2248098	*VDR*	65_5 & 65_6	rs1544410	*VDR*	65_5	*	*
rs2248098	*VDR*	65_5 & 65_6	rs7975232	*VDR*	65_5 & 65_6	*	n.s.
rs2248098	*VDR*	65_5 & 65_6	rs731236	*VDR*	65_5	*	*
rs2248098	*VDR*	65_5 & 65_6	rs739837	*VDR*	65_5 & 65_6	*	n.s.
rs7305032	*VDR*	65_6	rs2525044	*VDR*	65_6	*	*
rs7305032	*VDR*	65_6	rs7975232	*VDR*	65_5 & 65_6	*	*
rs7305032	*VDR*	65_6	rs739837	*VDR*	65_5 & 65_6	*	*
rs2525044	*VDR*	65_6	rs7975232	*VDR*	65_5 & 65_6	*	*
rs2525044	*VDR*	65_6	rs739837	*VDR*	65_5 & 65_6	*	*
rs1544410	*VDR*	65_5	rs7975232	*VDR*	65_5 & 65_6	*	n.s.
rs1544410	*VDR*	65_5	rs731236	*VDR*	65_5	*	*
rs1544410	*VDR*	65_5	rs739837	*VDR*	65_5 & 65_6	*	n.s.
rs7975232	*VDR*	65_5 & 65_6	rs731236	*VDR*	65_5	*	n.s.
rs7975232	*VDR*	65_5 & 65_6	rs739837	*VDR*	65_5 & 65_6	*	*
rs731236	*VDR*	65_5	rs739837	*VDR*	65_5 & 65_6	*	n.s.
rs11574114	*VDR*	None	rs2853563	*VDR*	None	*	*

If a SNP involved with significant r^2^ or D’ values is also associated with a significant CCC value, the BlocBuster network ID is given that contains an allele from that SNP. SNP, single nucleotide polymorphism; n.s., not significant.

### BlocBuster analysis of HapMap SNPs in the candidate genes

HapMap data did not contain many of the SNPs used to define network 65_2, but it did contain many additional SNPs in the four genes that contribute to 65_2. In general, when extended haplotypes exist, they are tagged by many SNPs. Therefore, we expected that some SNPs in the HapMap data should be able to identify at least some of the same components of 65_2 found in the analysis of our own data. We therefore performed a BlocBuster analysis on 571 HapMap SNPs in the four genes and their promoter regions that define 65_2 (*VDR*, *MC1R*, *SLC24A5*, and *SLC45A2*) in the four main HapMap populations (CEU, CHB, JPT, and YRI) with the most extensive SNP coverage. Although other HapMap populations are available, the overlap of SNP coverage with the four main populations results in a substantial decline of genetic resolution at the candidate genes. We therefore chose to maximize SNP coverage in order to enhance our genetic resolution for finding interlocus associations at the candidate loci.

Based upon 1000 permutation trials, the *CCC* threshold was set to 0.84 to eliminate false positives, as none appeared at this threshold level. Seven networks (Table 8) were identified in the combined four HapMap populations. Six of these networks correspond to single gene haplotypes, but one (84_2) is a multilocus network defined by 12 SNPs spanning two of the four genes found in 65_2 (Table 8), and includes two of the candidate SNPs in the original 65_2 network (*SLC24A5* rs1426654 and *SLC45A2* rs16891982).

**Table 8:** Networks identified by BlocBuster using a threshold of CCC ≥ 0.84 on the HapMap samples using 571 SNPs

Network	Gene	SNP ID	Allele	Chr	Position	Gene Location
84_1	*SLC45A2*	rs183671*	T	5	33964210	Intron 2
	*SLC45A2*	rs28777	C	5	33958959	Intron 3
	*SLC45A2*	rs35389	G	5	33954880	Intron 3
	*SLC45A2*	rs16891982*	C	5	33951693	Exon 5 (Phe374Leu)
	*SLC45A2*	rs35397*	G	5	33951116	Intron 5
	*SLC45A2*	rs35395*	T	5	33948589	Intron 5
	*SLC45A2*	rs35407	A	5	33946571	3′UTR
84_2	*SLC45A2*	rs183671*	G	5	33964210	Intron 2
	*SLC45A2*	rs28777	A	5	33958959	Intron 3
	*SLC45A2*	rs35389	A	5	33954880	Intron 3
	*SLC45A2*	rs16891982*	G	5	33951693	Exon 5 (Phe374Leu)
	*SLC45A2*	rs35397*	T	5	33951116	Intron 5
	*SLC45A2*	rs35395*	C	5	33948589	Intron 5
	*SLC45A2*	rs35407	G	5	33946571	3′UTR
	*SLC24A5*	rs12440301	G	15	48389924	5′UTR
	*SLC24A5*	rs12441154*	C	15	48390956	5′UTR
	*SLC24A5*	rs1834640	A	15	48392165	5′UTR
	*SLC24A5*	rs2675345	A	15	48400199	5′UTR
	*SLC24A5*	rs1426654	A	15	48426484	Exon 3 (Thr111Ala)
84_3	*SLC45A2*	rs35391	C	5	33955673	Intron 3
	*SLC45A2*	rs35390*	A	5	33955326	Intron 3
	*SLC45A2*	rs250417*	C	5	33952378	Intron 4
84_4	*VDR*	rs987849*	A	12	48254676	Intron 3
	*VDR*	rs11168268	A	12	48251812	Intron 3
84_5	*VDR*	rs4760655*	A	12	48294131	Promoter (intron 1a)
	*VDR*	rs10783219*	A	12	46581755	Promoter (intron 1a)
	*VDR*	rs7132324	C	12	46593576	Promoter
	*VDR*	rs10783219*	A	12	46581755	Promoter (intron 1a)
	*VDR*	rs7132324	C	12	46593576	Promoter
84_6	*SLC24A5*	rs1559857	G	15	48396808	5′UTR
	*SLC24A5*	rs2675346	C	15	48411821	5′UTR
	*SLC24A5*	rs2433354	C	15	48414969	Intron 2
	*SLC24A5*	rs2675347	A	15	48418645	Intron 2
	*SLC24A5*	rs2555364*	G	15	48419386	Intron 2
	*SLC24A5*	rs2675348	A	15	48420744	Intron 2
84_7	*SLC24A5*	rs2675345	G	15	48400199	5′UTR
	*SLC24A5*	rs2470102	G	15	484333494	Intron 8

SNP positions are indicated according to Genome Build 37.1. SNPs with an asterisk were excluded from the reduced networks used to test for latitudinal associations. SNP, single nucleotide polymorphism; Chr, chromosome; Phe, phenylalanine; Leu, leucine; UTR, untranslated region; Thr, threonine; Ala, alanine.

In order to examine associations with latitude, we required more populations than just the four used to define the networks. The remaining four HapMap populations (GIH, LWK, MKK, and TSI) were not scored for some of the SNPs, so reduced networks were created by dropping the unscored SNPs (Table 8). Reduced network 84_2R was now defined by seven SNPs spanning two of the genes in the original 65_2 network: *SLC45A2* and *SLC24A5*. Figure 5B shows a joint plot of the frequencies of individuals with 65_2 in our data and 84_2R in the HapMap data. As with 65_2R4, the Han Chinese and Japanese populations are extreme outliers, but when these two populations are excluded, the 65_2 and 84_2R points interpolate well and yield a highly significant linear regression against latitude with an R^2^ value of 0.85 (Table 9).

**Table 9:** The least-squares regressions of frequencies of individuals bearing the 84_2R network in the HapMap data set, and 84_2R plus 65_2 in the combined data set

Network	Genes	Intercept	Slope	Quadratic Term	R^2^
84_2R	*SLC24A5*, *SLC45A2*, *MC1R*	0.083	n.s.	n.s.	0.20
84_2R – As	*SLC24A5*, *SLC45A2*, *MC1R*	0.092	0.0037**	n.s.	0.87
84_2R – As + 65_2	*SLC24A5*, *SLC45A2*, *MC1R*, *VDR*	0.042	0.0180***	n.s.	0.84

** Significant with *P* ≤ 0.01, *** significant with *P* ≤ 0.001. n.s., not significant; – As, the Han Chinese and Japanese populations have been excluded.

### BlocBuster analysis of HapMap SNPs across the whole genome

BlocBuster was applied to all 1,115,561 autosomal SNPs that were common for the CEU, YRI, JPT, and CHB samples, and identified 21,748 allele-specific associations at the 0.84 threshold level. As expected, the number of associations increases with decreasing thresholds (382,001 at the 0.80 level, 86,461 at the 0.82 level, and 5042 at the 0.86 level). What is of more relevance here is that the proportion of associations that are interchromosomal decreases with increasing threshold level (32.0% at the 0.80 threshold, 11.5% at the 0.82 threshold, 2.9% at the 0.84 threshold, and 0.6% at the 0.86 threshold). As mentioned in the previous section, the permutation tests produced no false positives at the threshold of 0.84 and above, but false positives were encountered below this threshold. Given that unlinked SNPs tend to have lower *CCC* values overall, an increasing proportion of these false positives below the threshold of 0.84 fall into the interchromosomal category. On the other hand, exactly because unlinked SNPs tend to have lower *CCC* values than linked ones, going above the false positive threshold tends to preferentially eliminate the interchromosomal signals. These results show why it is important to determine the threshold at which false positives are eliminated or extremely rare; below that threshold there is much noise, particularly for interchromosomal contrasts, and above that threshold one rapidly eliminates almost all interchromosomal signals.

A total of 21,124 of the 21,748 allele-specific associations at the 0.84 threshold level were found between SNP alleles on the same chromosome, mostly tightly linked. A total of 627 associations involved SNPs on different chromosomes. Six of these involved the gene single-stranded DNA binding protein 4 (*SSBP4*) on chromosome 18 and its pseudogene on chromosome 19. This unusually strong association turned out to be an artifact of using identical primers for the gene and the pseudogene in the HapMap data. Excluding this artifact left 621 interchromosomal associations out of 21,742 allele-specific associations. The subset of the HapMap SNPs from our candidate genes revealed three interchromosomal associations at the 0.84 threshold and 38 intragenic ones, for a total of 41 *CCC* associations at or above 0.84. Hence, the remainder of the genome had 618 interchromosomal associations and 21,083 intragenic ones. The difference between the number of inter- *vs.* intrachromosomal associations in our four candidate genes *vs.* the remainder of the genome is highly significant (*P* < 0.0001) with a two-tailed Fisher’s Exact Test.

The 621 interchromosomal associations across the whole genome were due to two large networks, one with 561 interchromosomal associations (bloc1) and the other with 60 (bloc2). These two large networks mostly consisted of alleles that display a large frequency difference between at least two of the four HapMap populations (effectively three populations, as the Chinese and Japanese populations were very similar). The interchromosomal associations of our candidate genes were included in these large blocks as they also represented cases in which large frequency differences existed between at least two of the populations. These SNPs represent ancestry informative markers (AIMs) that are useful in admixture mapping (Bercovici *et al.* 2008). Since our candidate locus associations were absorbed into these large networks, we also calculated the frequencies of these blocks in the other HapMap populations given in Table 2, although with some SNPs dropping out of the blocks because they were not scored in all four of these additional populations. We then subjected these genome network frequencies to the same regressions against latitude as for our candidate gene network 84_2R. Unlike 84_2R (Figure 5 and Table 9), bloc1 did not display any significant quadratic regression with latitude, nor any regression at all when the two Asian populations were excluded, because bloc1 is found exclusively in the Chinese and Japanese population and has a network frequency of zero in all other HapMap populations. For bloc2, the quadratic coefficient was significantly different from zero, but yields a pattern of decreasing frequency with latitude followed by an increasing frequency with latitude (Figure S1) – a pattern not seen with any of our candidate networks. Excluding the two Asian populations did not change this pattern. Hence, at the whole genome level, multi-gene networks either had no or a qualitatively different type of association with latitude compared to our candidate gene networks.

## Discussion

### Interchromosomal LD

BlocBuster identified intragenic and interchromosomal LD, both in our own data and in the HapMap data. In contrast, the r^2^ and D’ analyses primarily identified just a subset of the intragenic associations revealed by BlocBuster (Figure 1 and Table 7) when, as in the BlocBuster analysis, the error rate is corrected for all pairs of SNPs. One reason for this discrepancy is allele specificity. For example, SNP rs3212357 in *MC1R* has significant LD (r^2^) with rs3212359, also in *MC1R* (Table 7). However, *CCC* makes it clear that the T allele at rs3212357 is associated with the C allele at rs3212359, whereas the C allele at rs3212357 is associated with the T allele at rs3212359, as previously shown (Stokowski *et al.* 2007; Mengel-From *et al.* 2009). Hence, there are two different allele-specific associations defined by these two SNPs that are part of two different haplotypes found in two different networks (65_3 and 65_7, Figure 1). This phase information is immediately available by using *CCC*, but is not directly apparent with the scalar LD measures. Information about potential natural selection is also lost by r^2^ and D’ relative to *CCC*. One of the phased *MC1R* haplotypes associated with the SNP pair rs3212357 and rs3212359 has a significant latitudinal cline, but the other does not (Table 3 and Table 4). By pooling these two different haplotypes into a single scalar measure, r^2^ obscures their different responses to the latitudinal gradient, and D’ failed to detect any association at all between these SNPs. Consequently, *CCC* returns more biological information about the nature of the association even when r^2^ identified the same pair of SNPs.

A second reason for this discrepancy between BlocBuster and the scalar LD analyses is that none of the eight intergenic associations detected by *CCC* (Figure 1) were detected with r^2^ and D’ (Table 7), even though the disequilibrium statistics were adjusted to an overall type I error of 0.05, whereas *CCC* was adjusted to a more stringent level of 1.69 × 10^−5^ per element or the 1% level for false discovery including all genes and SNPs. We also failed to find any significant interchromosomal LD (D’ and r^2^) after multiple testing correction, even when we focused the statistical power upon the interchromosomal SNP pairs by permuting intact multiple-SNP genotypes at each gene. These results show the importance of allele specificity in defining these networks and dealing with genetic heterogeneity among allelic-specific combinations.

We now examine possible explanations for the intergenic associations detected by BlocBuster.

#### The interchromosomal allelic associations are false positives:

A simple explanation for the interchromosomal allele-specific associations identified by *CCC* and BlocBuster is that they are all false positives. However, the permutation test of the null hypothesis of no association indicates an expected number of 0.137 false positives per trial (both intra- and interchromosomal) on all 2016 SNP pairs, whereas the observed number of just the interchromosomal associations above the 0.65 threshold was eight (Figure 1) – a 63-fold excess above the expected number of false positives for all pairs. Moreover, using the all genes/all SNPs interpretation of the false-discovery rate of the permutation test results, any result with more than one *CCC* value ≥ 0.65 can be rejected as due to chance at the 1% level of significance. Hence, it is extremely unlikely that all eight significant interchromosomal pairs were false positives.

Another powerful way to eliminate false positives is to replicate the results in a second data set. Many, but not all, of the HapMap populations (Table 2) come from geographical regions that are nearby to the geographical regions in our sample (Table 1), but even in those cases the HapMap populations do represent an independent sample from that region. Although the HapMap data only partially overlaps the SNPs in our study, applying *CCC* and BlocBuster to the HapMap data also revealed significant interchromosomal disequilibrium between SNP pairs involving two of the same genes and two of the shared SNPs found in our multilocus network 65_2 (Table 8). Not only were these interchromosomal disequilibria replicated, but the latitudinal associations of the resulting multilocus networks were statistically indistinguishable for both data sets (Figure 5 and Table 6). This replication in a second data set makes it extremely unlikely that all the interchromosomal disequilibria detected and their latitudinal associations were false positives.

We conclude that these are true signals of interchromosomal association because of the results of the permutation testing and the ability to replicate in a second dataset. The question then becomes, what are the causes of this signal?

#### The interchromosomal allelic associations are neutral artifacts of population structure and/or history:

We were specifically interested in allele-specific associations with our candidate genes across populations, rather than within populations, in order to investigate possible selective responses to variation in latitude. Accordingly, we applied *CCC* and BlocBuster to the total sample in order to find allele-specific associations that were found across multiple populations. The danger with using global LD instead of within-population LD is that any evolutionary force that induces allele frequency differences among local populations will induce LD in the pooled population (Li and Nei 1974; Templeton 2006, pg. 74). The balance between local genetic drift and gene flow, or historical effects such as past bottlenecks or founder events, can all generate LD even between unlinked loci at the total population level. A simple yet effective control that makes no assumptions concerning population structure or demographic history is to sample randomly chosen SNPs and test if the candidate SNPs are outliers with respect to the random SNPs (Fraser 2013). Our set, consisting of more than 1,000,000 SNPs, confirms that the BlocBuster networks containing genetically unlinked loci are highly significant outliers. Fraser (2013) emphasizes that the case for local adaptation is greatly strengthened by an overlap of genetic outlier status with environmental variation related to the genes’ functions. Using this overlap criterion, the networks of SNP alleles that we identified imply the action of natural selection and are inconsistent with just random drift or demographic history. In addition, of the 621 interchromosomal associations found across the whole genome, 571 of them defined a massive network that had no significant association with latitude, and the remaining 60 defined a network that had a decreasing followed by increasing association with latitude (Figure S1), completely unlike our candidate gene networks. Consequently, the latitudinal associations found with 65_1, 65_2, or 84_2 cannot be explained by a genomic background effect, which further strengthens an adaptive explanation.

The LD created by population structure and demographic history should also influence scalar LD measures, but no interchromosomal scalar LD is found (Table 7), implying that allele specificity was a critical feature in the interchromosomal LD found by BlocBuster. The LD that arises from the balance of genetic drift *vs.* gene flow or from demographic history is not, in general, allele specific (Templeton 2006, pgs. 94–95). The importance of allele specificity in the detected interchromosomal LD further undermines the hypothesis that these multilocus patterns arose from neutral population structure or historical processes.

As reviewed in Templeton (2015), the genetic, ancient DNA, paleoclimatic, paleontological, and archeological evidence indicate that there was a major population expansion out of sub-Saharan Africa, starting ∼130,000 years ago, into Northern Africa, the Levant, and the Arabian Peninsula, which continued across the southern portions of Eurasia and eventually reached Eastern Asia. This expanding population of African origin engaged in limited admixture with Eurasian populations. Then, starting ∼50,000 years ago, there was an expansion out of Southern Eurasia into Northern Eurasia, and more recently an expansion of human populations into the Americas, going from the north to the south. Although there was speculation that population expansion along a particular gradient could create neutral genetic associations with that gradient, detailed analyses and simulations reveal that just the opposite is true (Frichot *et al.* 2015), with neutral associations actually being much less likely along the expansion gradient than along any other direction. Since our multilocus networks show significant alignment with latitude, human population expansions *per se* are unlikely to have been the cause of this association.

In light of the above, natural selection is strongly indicated as having shaped the observed association and patterns in our candidate loci. The inference of selection is not surprising, given that earlier studies have also indicated that positive natural selection has operated upon many of these candidate genes to result in a latitudinal cline in human skin pigmentation (Jablonski and Chaplin 2010, 2013; Beleza *et al.* 2013a). The skin color genes are well known to influence vitamin D generation by the capacity of melanin to absorb UVB energy, and once vitamin D is produced, its nuclear receptor VDR plays a central role in the signaling of vitamin D to influence a variety of important health-related traits (Fang *et al.* 2006; Uitterlinden *et al.* 2006; Azad *et al.* 2012; Bikle 2012; Disanto *et al.* 2012; Jovicic *et al.* 2012; Chaplin and Jablonski 2013; Christakos *et al.* 2013; Mao and Huang 2013; Tiosano *et al.* 2013; Abd-Allah *et al.* 2014; Borella *et al.* 2014; Cantorna and Waddell 2014; Cutolo *et al.* 2014; Laplana *et al.* 2014; Liu *et al.* 2014; Mann *et al.* 2014; Rolf *et al.* 2014). Hence, there is also much potential for strong selection at the *VDR* locus. Another selective force that could result in clines is the strong relationship of vitamin D to immune functions. Specifically, tuberculosis may have been a selective agent on vitamin D metabolism as humans migrated into Eurasia’s higher latitudes (Kappelman *et al.* 2008). Interestingly, the skin color gene *MC1R* has also been shown to possess an immunoregulatory activity (Bohm *et al.* 2005). Hence, the potential for selection is high in our candidate genes.

Consistent with previous studies indicating clinal selection on several skin color genes, BlocBuster identified three allele networks within our candidate loci (Figure 1) that had significant latitudinal clines. One of these was the single locus haplotype 65_3 in the *MC1R* gene. *MC1R* had previously been interpreted as under relaxed selection and not positive selection at the higher latitudes (Jablonski and Chaplin 2013). This haplotype does show a nonlinear association with latitude that is due to the sub-Saharan populations, consistent with differing types of selection in the tropics *vs.* higher latitudes (Figure 2 and Table 4). However, there is a significant linear cline when just the higher latitudes are used (Table 4), which is inconsistent with the inference of only relaxed selection at this locus in the higher latitudes.

The other two networks in Figure 1 that showed significant latitudinal associations were 65_1 and 65_2, both multilocus clines consisting of markers in the *VDR* and skin color genes (Figure 3 and Figure 4). The multilocus complexes show significant, allele-specific clines with latitude, a proxy for the environmental variable of UVR intensity that presumably drives natural selection at these genes (Figure 3, Figure 4, and Figure 5). Hence, the coordinated shifts in allele frequencies at multiple loci are well explained by an environmental variable that directly relates to the function of these candidate genes. De Mita *et al.* (2013) showed that correlations with an environmental variable are a powerful method of detecting selection, but can also lead to false-positives when between population correlations exist unless the underlying correlation structure is taken into account. This underlying correlation structure should influence the entire genome. Our genomic control showed that the multilocus complexes identified with our candidate genes are highly significant outliers within the autosomal genome, indicating that a common underlying correlation structure is not the source of our multilocus responses to latitude. Moreover, the multilocus networks found at the total autosomal genome level did not display the same correlation structure with latitude as our candidate gene networks. All of these results strengthen the interpretation of a response to selection by our candidate genes. However, a multilocus response to selection can arise from single-locus responses to a common environmental variable, as we now discuss.

#### The interchromosomal allelic associations arose from parallel responses to natural selection:

Because all of the candidate genes that we studied could be related to a single environmental variable (UVB and its proxy latitude), the *CCC* allelic correlations between our candidate genes could be attributed to parallel single-locus selection on the different genes due to a common environmental influence; that is, two or more nonepistatic loci may show similar clines to the same environmental variable, and the resulting parallel single-locus responses create a correlated, clinal multilocus response.

Figure 2, Figure 3, Figure 4, and Table 4 reveal that many of the single-locus haplotypes and SNPs of multilocus networks show significant clines with latitude, and this could induce parallel clinal correlations between these loci. However, most of these single locus clines were not parts of the same multilocus complex or even part of any multilocus complex (*e.g.*, 65_3 in Figure 2 and Table 4). Hence, parallel clinal responses to natural selection at single loci are not sufficient to lead to clinal *CCC* correlations between genes.

Many of the single locus elements within a multilocus network did not display parallel clinal behavior, even though the multilocus networks containing them did show clinal behavior. Network 65_1 consists of three single locus components: a haplotype in the skin color gene *TYRP1*, a single SNP in the skin color gene *TYR*, and a haplotype in the *VDR* promoter. By itself, the *VDR* promoter haplotype shows no significant regression with latitude, the *TYRP1* haplotype shows a significant linear decline in frequency with increasing latitude, and the *TYR* SNP shows a weak, but significant, linear increase in frequency with increasing latitude (Figure 3 and Table 4). Hence, the single locus components of 65_1 each display a different association with latitude, even though network 65_1 shows a significant linear decline with latitude (Table 4). There are no parallel responses to selection by any of the single locus components in network 65_1.

The multilocus network 65_2 contains a three-SNP haplotype in the *VDR* promoter region (haplotype 70_4) that shows a dramatic in- *vs.* out-of-Africa frequency pattern, with a frequency of 0.2 in sub-Saharan Africa, and an average frequency of 0.9 with no latitudinal association outside of Africa (Figure 4 and Table 4). Similar in- *vs.* out-of-Africa frequency differences, albeit weaker, are shown by SNPs in two unlinked skin color genes: *MC1R* SNP rs2228478 and *SLC24A5* SNP rs1426654 (Figure 4 and Table 4). For all five SNPs showing this in- *vs.* out-of-Africa pattern, it is the derived allele that has risen to high frequency outside of Africa (ancestral alleles are given in dbSNP, http://www.ncbi.nlm.nih.gov/projects/SNP/index.html), further supporting the idea that selection drove all of these derived alleles to high frequency outside of Africa. These similar patterns could account for some of the *CCC* associations in network 65_2. Another *VDR* promoter, SNP rs4073729, shows a significant nonlinear regression with latitude due to a lower frequency in sub-Saharan Africa *vs.* the remaining populations, but in this case there is a weak but significant decline in frequency outside of Africa with increasing latitude (Figure 4 and Table 4). The final two components of network 65_2, *MC1R* SNP rs3212369 and *SLC45A2* SNP rs16891982, show significant linear clines with frequency increasing with increasing latitude (Figure 4 and Table 4). Hence, there are at least three different patterns of association with latitude among the components of network 65_2, so parallel responses to natural selection cannot explain all the *CCC* associations found in this network.

Collectively, the patterns shown by the single locus components of 65_1 and 65_2, and by SNPs in 65_3, reveal that parallel patterns with latitude are neither necessary nor sufficient for membership in a multilocus network detected by BlocBuster. Parallel responses to an environmental gradient can explain some but not all of the associations uncovered by *CCC*.

#### The interchromosomal allelic associations arose from coadaptation:

The concept of coadaptation was introduced by Dobzhansky (1948) in the context of a geographically widespread species undergoing local adaptation that depended upon fitness interactions between specific alleles at different loci. Wallace (1968) defined coadaptation as “Any adjustment of the frequencies of alleles at one locus in response to changes of those at another” where the adjustment is effected through natural selection acting through fitness epistasis between loci. Therefore, coadaptation could cause an association of specific alleles at different loci across local populations.

Coadaptation does not depend upon the allelic associations between genes having correlated parallel responses to selection. For example, selection could favor low frequencies of an allele at a major gene in some ranges of environments, and high frequencies in another range, with no clinal behavior within these environmental ranges, such as observed with the *VDR* haplotype 70_4. Epistatic modifiers of this major gene could then fine-tune the local adaptation and show clinal behavior. Note that the selection on the modifiers in a local population is on a relatively constant genetic background defined by the major gene that first responded to local conditions. In such a situation, the Mendelian epistasis between the modifiers with a major locus is transformed primarily into “additive genetic variance” in the quantitative genetic sense, with little or no additive variance at the major locus (Templeton 2006, pgs. 330–337; Mäki-Tanila and Hill 2014). Since the response to selection is determined by the additive genetic variance, the Mendelian epistasis in such a system translates primarily as individual loci responding additively to selection at any given time with little to no within local-population LD, particularly for unlinked loci (Templeton 2006, pgs. 409–412). Hence, the LD associated with coadaptation is found primarily at the global population level due to the mutual adjustment of allele frequencies. We therefore chose to study this global LD rather than within local-population LD, as done by Daub *et al.* (2013) in looking for coadaptation to pathogens within local human populations. Such within population LD may sometimes exist in coadapted complexes, but it is not necessary nor is it demanded by the original definition of coadaptation. Therefore, our focus was on global LD with appropriate genomic controls to address the effects of population structure and demographic history.

Although parallel responses to selection are not required for the components of a coadapted complex, Mendelian epistasis is necessary. The candidate trait for being the direct target of selection has often been skin pigmentation (*e.g.*, de Magalhães and Matsuda 2012; Rees and Harding 2012). In this regard, skin pigmentation variation has been shown to be strongly influenced by *VDR* and skin color genes with an epistatic genetic architecture (Pośpiech *et al.* 2014). Genetic variation at the *VDR* locus has been associated with important health traits in a manner suggestive of epistasis with other loci because of interactions with “ethnicity” (Mao and Huang 2013; O’Neill *et al.* 2013). Accordingly, these genes are biologically plausible candidates for producing coadapted complexes through epistatic selection on skin color and/or immune functions.

Although the single locus components of 65_1 and 65_2 show highly diverse latitudinal patterns, when brought together into a multilocus network both networks show strong linear regressions with latitude (Figure 3 and Figure 4). The whole is clearly not the sum of the parts for these multilocus complexes, consistent with coadaptation but not parallel adaptation. Moreover, even when the individual genes that show strong clinal behavior with latitude are excluded (65_2R4), the remaining portions of these complexes still display significant clinal associations with latitude (Figure 5A and Table 6). The whole being greater than the sum of the parts is strongly reinforced by multilocus network 84_2. The most extreme geographical differentiation for any of the single locus elements is the *VDR* haplotype 70_4, which has very low frequencies in sub-Saharan Africa and very high frequencies elsewhere in our sample. Because of missing SNPs in the HapMap data, the *VDR* promoter was not included in the multilocus complex 84_2, yet this reduced complex still displays virtually identical behavior to 65_2 across latitude (Figure 5B). Once again, the whole is not just the sum of the parts.

Overall, many of the significant multilocus correlations detected by *CCC* are more consistent with coadaptation than with parallel adaptation, although others (such as the in- *vs.* out-of-Africa SNPs in network 65_2) could be explained by parallel selective responses. Hence, our results most likely reflect a combination of parallel selective responses and coadaptation between loci with known epistasis for a trait under selection.

### The timing of selection on multilocus networks

Insight into the timing of selection for the 65_2 complex is provided by Beleza *et al.* (2013b). They estimated the timing of the selective sweeps in European populations at the *SLC24A5* and *SLC45A2* loci and included the same SNPs used in our study to mark these genes, both of which are part of the 65_2 complex (Table 3). Their estimate of the timing of the European selective sweep was 11,000 to 19,000 years ago. Similarly, the 95% confidence interval for the onset of positive selection at *TYRP1*, a component of 65_1, in Northern Europeans is 8350 to 18,590 years ago (Chen *et al.* 2015), indicating that components of both detected multilocus complexes are of comparable and relatively recent origin in human evolution. Indeed, an analysis of the genome of a 7000-year-old European indicates that light skin was not yet ubiquitous in Mesolithic Europe (Olalde *et al.* 2014), and light skin color in Europe has been strongly favored over the last 5000 years (Wilde *et al.* 2014).

To gain insight into the age of the VDR haplotype 70_4 component of the 65_2 complex, we examined the Neandertal and Denisovan genome databases (http://neandertal.ensemblgenomes.org/, and http://www.eva.mpg.de). Neither had the 65_2 network, including the VDR haplotype 70_4. This implies that the 65_2 network and the 70_4 haplotype did not arrive in Northern Eurasia until sometime after 50,000 years ago.

### The role of VDR

Both of the multilocus complexes identified by BlocBuster involve the *VDR* gene. Despite the central role of VDR in vitamin D utilization and the evidence for selection at VDR binding sites (Ramagopalan *et al.* 2010), there has been little evidence for selection on genetic variants at the *VDR* locus. BlocBuster identified five extended haplotypes in the *VDR* gene, but only one, haplotype 70_4, has a significant geographical association: being rare in sub-Saharan Africa and East Asia, and very common elsewhere. This *VDR* haplotype is also a component of the strongest multilocus complex cline associated with network 65_2, which varies almost over the entire 0–1 frequency range with latitude. This *VDR* haplotype contains SNPs in the promoter region, including rs11568820 that is located in the site interacting with the intestinal-specific transcription factor Cdx2, which is associated with bone mineral density (BMD) and fracture risk. The G allele at this SNP is associated with lower BMD (Arai *et al.* 2001) and the A allele is associated with lower fracture risk (Fang *et al.* 2003; Uitterlinden *et al.* 2006). The G allele has a diminished transcriptional activity (Arai *et al.* 2001; O’Neill *et al.* 2013), with sub-Saharan Africans having higher VDR protein levels (Beleza *et al.* 2013a). The sharp increase in G allele frequency in most populations outside of sub-Saharan Africa suggests that the frequency of the less transcriptionally active *VDR* promoter haplotype was favored as humans ventured out of Africa, while other genes associated with skin color adapted to a progressively lighter skin on the genetic background of this less active *VDR* promoter. However, the HapMap data indicate that Han Chinese and Japanese mostly have the more active promoter and do not have the 65_2 multilocus complex, an observation that is consistent with previous analyses that these far East-Asian populations achieved their light skins in a convergent but genetically distinct manner to Europeans (Norton *et al.* 2007; Edwards *et al.* 2010; Yamaguchi *et al.* 2012). Interestingly, our Native American sample does not cluster with the Chinese and Japanese populations, but rather fits the pattern defined by our samples from Africa, the Middle East, Europe, and India (Figure 5). This result is consistent with a genome sequenced from a 24,000-year-old Siberian that indicated that between 14–38% of Native American ancestry derives from this Siberian population, which in turn is genetically related to modern-day Western Eurasians with no close affinity to East Asians (Raghavan *et al.* 2014).

The *VDR* promoter haplotypes appear to have interacted with skin color genes to produce fine-scale adaptation to higher latitudes and decreasing UVB irradiation in a coadapted fashion. *VDR* is the only locus that was a component of both of the multilocus complexes detected by BlocBuster with our data, and both of these complexes showed significant latitudinal clines (Table 4). Yet, all of the haplotypes within *VDR*, including those that were components of the multilocus complexes, displayed either no latitudinal association at all, or an in- *vs.* out-of-Africa pattern when examined in isolation, although one *VDR* SNP had a weak cline outside of Africa. Moreover, unlike the skin color genes, the previous literature does not report signatures of selection at *VDR*. This result is not unexpected in light of the empirical studies on coadaptation in *Drosophila mercatorum* (Templeton *et al.* 1976). The results of these experiments showed “that loci may appear neutral on the margin but still be involved in a highly selected gene complex,” leading to the warning “that selective neutrality may arise as an artifact in a coadapted genetic complex if the genetic markers used do not identify the unit of selection” (in this case, the multilocus complex). *VDR* appears to be such a gene that is subject to strong selection, but only in the context of its interactions with other genes. This also illustrates the limitation of inferring selection on the basis of metrics applicable to only one gene or one small genomic region at a time – the current norm for selection scans of the genome. Such scans for selection will undoubtedly miss many of the adaptive processes that occurred in human evolution. BlocBuster helps fill that gap.

### Properties of BlocBuster

BlocBuster is a novel approach for identifying regions of the genome that are correlated at the population level in an allele-specific fashion, either genetically unlinked regions or within contiguous regions along a chromosome. BlocBuster successfully identified single-locus haplotypes and multilocus complexes that display significant frequency regressions with latitude, indicative of local adaptation to UVR. The significant *CCC* associations between genetically unlinked loci were invisible to the analyses using r^2^ and D’, two standard scalar measures of LD (Table 7), even when statistical power was enhanced by limiting the multiple testing correction to only interchromosomal pairs. These results demonstrate that BlocBuster is a versatile tool for detecting allelic associations both in small genomic regions and between unlinked loci. Our genome-wide survey of the HapMap data indicated that multilocus complexes are rare, but still indicated several potential multilocus complexes in the human genome that will be the focus of future work. Hence, BlocBuster and *CCC* add a new method of looking for associations between SNPs and for studying natural selection at the multilocus level.

## Supplementary Material

Supplemental Material
