# Supplementary material for: Latitudinal Clines of the Human Vitamin D Receptor and Skin Color Genes
Source: G3 (Bethesda). 2016 Feb 26;6(5):1251–66. doi: 10.1534/g3.115.026773 (PMC4856077; doi:10.1534/g3.115.026773)
Supplement: Supplemental Material [file supp_6_5_1251__index.html]

Latitudinal Clines of the Human Vitamin D Receptor and Skin Color Genes — Supplemental Material 

# Latitudinal Clines of the Human Vitamin D Receptor and Skin Color Genes

## Supplemental Material for Tiosano *et al.*, 2016

**Files in this Data Supplement:**

- Figure S1 - A plot of the points and least-squares regression (red line) of the frequency of the genome wide Bloc2 from the HapMap data versus latitude. (.pdf, 20 KB)
- Table S1 - SNPs performed using TaqMan assays on OpenArray platform (Applied Biosystems). (.pdf, 38 KB)
- File S1 - The SNP genotypes used in the study. (.xls, 273 KB)
