# Supplementary material for: Latitudinal Clines of the Human Vitamin D Receptor and Skin Color Genes
Source: G3 (Bethesda). 2016 Feb 26;6(5):1251–66. doi: 10.1534/g3.115.026773 (PMC4856077; doi:10.1534/g3.115.026773)
Supplement: Supplemental Material [file supp_g3.115.026773_FigureS1.pdf]

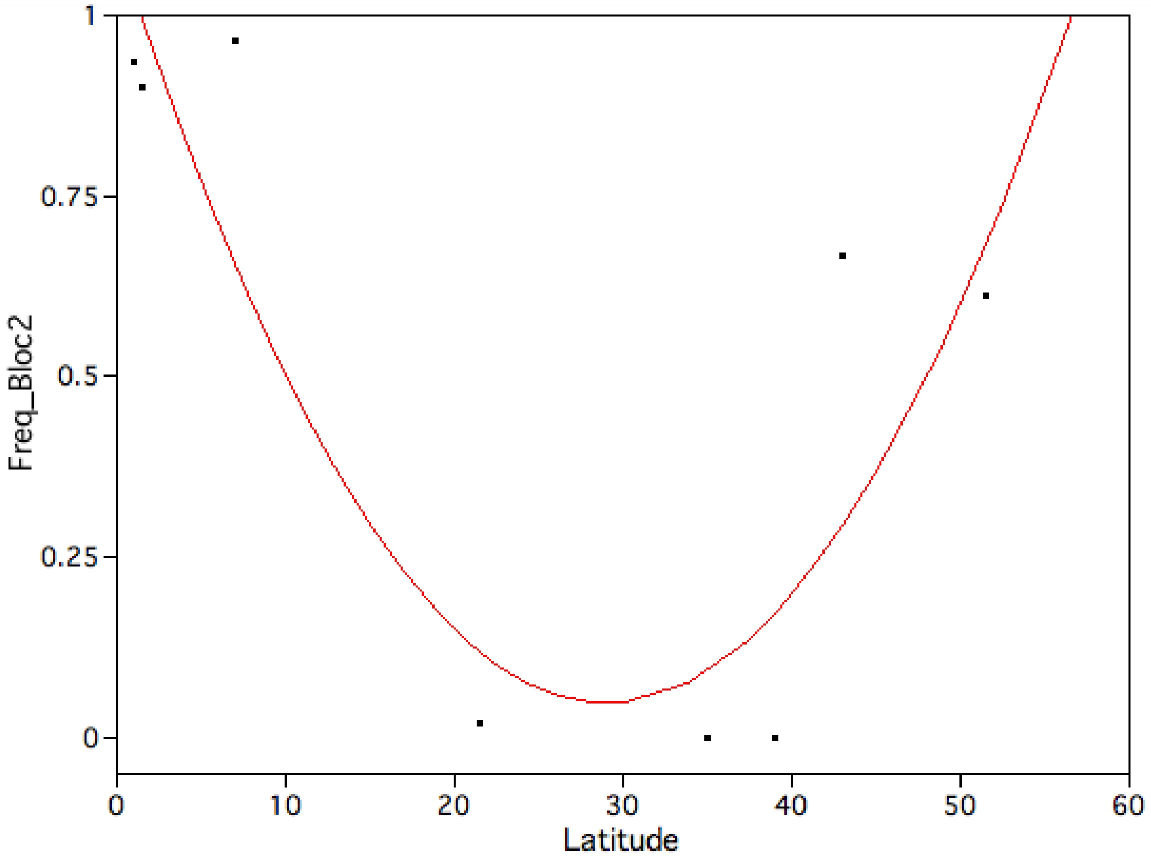

Figure S1. A plot of the points and least-squares regression (red line) of the frequency of the genome wide Bloc2 from the HapMap data versus latitude.
