# Supplementary material for: Latitudinal Clines of the Human Vitamin D Receptor and Skin Color Genes
Source: G3 (Bethesda). 2016 Feb 26;6(5):1251–66. doi: 10.1534/g3.115.026773 (PMC4856077; doi:10.1534/g3.115.026773)
Supplement: Supplemental Material [file supp_g3.115.026773_TableS1.pdf]

Table S1. SNPs performed using TaqMan assays on OpenArray platform (Applied Biosystems). SNP positions are indicated according to Genome Build 37.1

Gene	TaqMan assay name	Number dbSNP	Nucleotides	Position	Gene Location
**TYR-1**	C___8362862_10	rs1042602	C / A	88,911,696	Ser192Tyr
**TYRP1-1**	C___1911864_20	rs1408799	T / C	12,672,097	5'UTR
**TYRP1-2**	C___3119210_10	rs2733832	C / T	12,704,725	Intron 5
**OCA2-1**	C___8866200_10	rs1800407	G / A	28,230,318	Arg419Gln
**OCA2-2**	C___8866240_10	rs1800414	A / G	28,197,037	His615Arg
**SLC45A2-1**	C___2390575_1_	rs26722	G / A	33,963,870	Glu272Lys
**SLC45A2-2**	C___2842665_10	rs16891982	C / G	33,951,693	Phe374Leu
**SLC24A5**	C___2908190_10	rs1426654	A / G	48,426,484	Thr111Ala
**KITLG**	C__26988870_10	rs12821256	T / C	89,328,335	5'UTR
**MC1R-1**	C__27982534_20	rs3212357	T / C	89,984,604	5'UTR
**MC1R-2**	C__60403717_10	rs34057592	C / T	89,985,051	5'UTR
**MC1R-3**	C__32390814_10	rs3212379	C / T	89,985,131	5'UTR
**MC1R-4**	C___2033408_20	rs3212359	C / T	89,985,177	5'UTR
**MC1R-5**	C__27841133_10	rs76337330	A / G	89,985,311	5'UTR
**MC1R-6**	C___2033406_1_	rs3212363	A / T	89,985,441	5'UTR

**MC1R-7**	C___7519054_10	rs1805005	G / T	89,985,844	Val60Leu
**MC1R-8**	C__60403720_10	rs34090186	G / A	89,985,866	Arg67Gln
**MC1R-9**	C___7519055_20	rs1805006	C / A	89,985,918	Asp84Glu
**MC1R-10**	C__60403722_10	rs34540312	G / A	89,985,931	Gly89Arg
**MC1R-11**	C___2033405_20	rs2228479	G / A	89,985,940	Val92Met
**MC1R-12**	C__60403725_10	rs33932559	T / C	89,986,025	Ile120Thr
**MC1R-13**	C__27541634_10	rs11547464	G / A	89,986,09	Arg142His
**MC1R-14**	C___2033404_30	rs1805007	C / T	89,986,117	Arg151Cys
**MC1R-16**	C___7519060_20	rs885479	G / A	89,986,154	Arg163Glu
**MC1R-17**	C__25647438_10	rs34612847	C / T	89,986,170	Ile168Ile
**MC1R-18**	C__60403730_10	rs34209185	C / T	89,986,215	Tyr183Tyr
**MC1R-19**	C__32390806_20	rs3212366	T / C	89,986,252	Phe196Leu
**MC1R-20**	C__60403731_20	rs34564466	G / A	89,986,299	Leu211Leu
**MC1R-21**	C__25647439_10	rs34490506	C / T	89,986,383	Gly239Gly
**MC1R-22**	C__27860175_10		A / C	89,986,445	His260Pro
**MC1R-23**	C__25647446_10	rs3212367	C / T	89,986,566	Phe300Phe
**MC1R-24**	C___2033403_10	rs2228478	A / G	89,986,608	Thr314Thr
**MC1R-25**	C___2033402_10	rs3212369	A / G	89,986,760	3'UTR
**MC1R-26**	C__25958294_10	Rs 143513600	A / G	89,986,957	3'UTR
**MC1R-27**	C__25960110_10	rs3212371	A / G	89,987,201	3'UTR
**VDR-1**	C__27909097_10	rs4237856	G / T	48,338,050	Promoter
**VDR-2**	C__27909106_10	rs4073729	C / T	48,337,069	Promoter
**VDR-3**	C___2880808_10	rs11568820	G / A	48,302,545	Promoter (Cdx2)

**VDR-4**	C__27904554_10	rs4760658	T / C	48,296,486	Promoter (Intron 1a)
**VDR-5**	C___2880803_10	rs10783219	T / A	48,295,488	Promoter (Intron 1a)
**VDR-6**	C__44841112_10	rs11168293	C / A	48,293,716	Promoter (Intron 1a)
**VDR-7**	C__12060037_10	rs10875695	G / T	48,293,037	Promoter (Intron 1a)
**VDR-8**	C__12060038_10	rs7302235	A / G	48,292,838	Promoter (Intron 1a)
**VDR-9**	C__15823889_10	rs2853564	C / T	48,278,487	Promoter (Intron 1a)
**VDR-10**	C__12060044_1_	rs1989969	T / C	48,278,010	Promoter (Intron 1a)
**VDR-11**	C___3290655_1_	rs2238136	G / A	48,277,713	Promoter (Intron 1a)
**VDR-12**	C__12060045_20	rs2228570	T / C	48,272,895	Exon2 (Met1Thr) [FokI]
**VDR-13**	C___3290647_10	rs3782905	C / G	48,266,167	Intron 2
**VDR-14**	C___3290638_1_	rs2238138	C / T	48,264,493	Intron 2
**VDR-15**	C__30742507_10	rs11574070	C / T	48,257,895	Intron 3
**VDR-16**	C___3290631_1_	rs2239180	G / C	48,256,046	Intron 3
**VDR-17**	C___3290630_1_	rs2248098	T / C	48,253,356	Intron 3
**VDR-18**	C__30742483_10	rs7305032	C / T	48,249,860	Intron 5
**VDR-19**	C__16174104_10	rs2239183	G / T	48,244,660	Intron 6
**VDR-20**	C__30742462_10	rs12314197	T / C	48,242,722	Intron 6
**VDR-21**	C__16031766_10	rs2525044	T / C	48,242,256	Intron 6
**VDR-22**	C___8716062_10	rs1544410	G / A	48,239,835	Intron 8 [BsmI]
**VDR-23**	C__30742443_10	rs11574114	G / A	48,238,883	Intron 8
**VDR-24**	C__28977635_10	rs7975232	G / T	48,238,837	Intron 8 [ApaI]
**VDR-25**	C___2404008_10	rs731236	T / C	48,238,757	Exon 9 (Ile352Ile) [TaqI]
**VDR-26**	C___2404007_10	rs739837	C / A	48,238,221	3'UTR

**VDR-27**	C___2404006_10	rs3847987	G /T	48,238,068	3'UTR
**VDR-28**	C__15823846_10	rs2853563	G / A	48,235,738	3'UTR
